# Next generation automated traceless cell chromatography platform for GMP-compliant cell isolation and activation

**DOI:** 10.1038/s41598-022-10320-x

**Published:** 2022-04-21

**Authors:** Sabine Radisch, Mateusz P. Poltorak, Michaela Wagner, Vlad Cletiu, Christian Radisch, Irina Treise, Steffi Pann, Alexis Weigt, Sophie Artner, Stefan Dreher, Fabian Fechner, Bojana Borjan, Simon P. Fraessle, Manuel Effenberger, Eileen Benke, Gottfried Navratil, Norbert Hentschel, Dirk H. Busch, Thomas Schmidt, Christian Stemberger, Lothar Germeroth

**Affiliations:** 1 Juno Therapeutics GmbH, Bristol-Myers Squibb Company, Grillparzerstr. 10, 81675 Munich, Germany; 2 Institute for Medical Microbiology Immunology and Hygiene, Technical University of Munich, Munich, Germany

**Keywords:** Cancer immunotherapy, T cells, CD4-positive T cells, CD8-positive T cells, Immune cell isolation

## Abstract

Large-scale target cell isolation from patient blood preparations is one of the critical operations during drug product manufacturing for personalized cell therapy in immuno-oncology. Use of high-affinity murine antibody coated magnetic nanoparticles that remain on isolated cells is the current standard applied for this purpose. Here, we present the transformation of previously described technology — non-magnetic immunoaffinity column chromatography-based cell selection with reversible reagents into a new clinical-grade cell isolation platform called Automated Traceless Cell affinity chromatography (ATC). ATC is a fully closed and GMP-compliant cell selection and manufacturing system. Reversibility of reagents enables (sequential) positive cell selection, optionally in combination with depletion columns, enabling capture of highly specific cell subsets. Moreover, synergy with other Streptamer-based technologies allows novel uses beyond cell isolation including integrated and automated on-column target cell activation. In conclusion, ATC technology is an innovative as well as versatile platform to select, stimulate and modify cells for clinical manufacturing and downstream therapies.

## Introduction

Cell selection is a prerequisite in cell-based immune therapies to achieve desired cell product compositions. It is mostly done through upfront selection prior to downstream cell product processing or by in-process purification. It can be limited to simply separating leukocytes from blood by using density gradients or executed with highly sophisticated enrichment of a specific cell subpopulation applying isolation reagents targeting cell specific markers or biological traits^1–4^. As expected, more precise cell subset selection permits better definition, predictability and control of the final cell product characteristics, but may dramatically increase selection complexity including multiple non-automated handling steps as well as time and cost of cell manufacturing. Additionally, an increase of individual operation steps required to enrich for target populations will directly translate into cell loss from any given starting material. These hindrances can be amplified by the selection methodology itself impacting downstream manufacturing procedures. Therefore, process automation and novel selection technologies are needed to overcome these limitations.

Currently, researchers are exploring the utilization of many types of immune cells to combat diseases with a great focus on oncogenic malignancies pioneered by impressive results obtained with chimeric antigen receptor (CAR) modified T cells for hematologic indications^5–7^. Today, all commercially available CAR T cell products are exclusively T cell derived and the majority of cell isolation technologies are designed to enrich for either CD3^+^ or CD4^+^ and CD8^+^ T cells^8^. The need for selection and enrichment of T cells with certain phenotypic and functional attributes is important for the success of the multi-step manufacturing of complex cell products. Upfront selection of target cells reduces the complexity of common blood-derived patient material and provides a robust and consistent intermediate, lowering risks caused by unwanted cell contaminants. In addition to upfront isolation, in-process purification of desired cell populations, especially in context of current sophisticated gene editing and engineering approaches, has the potential to refine the final drug product and enhance functional and safety attributes. The functionality of purified cells can be further preserved by appropriate chromatographic selection technologies that allow quantitative removal of process reagents or byproducts from the cells^9,10^.

Hence, cell purification technologies in combination with next generation bioengineering approaches provide an attractive integrated platform. Such platforms enable a closed and automation-controlled system that improves the turn-around time and reduces the manufacturing costs simultaneously minimizing hands-on complexity. Currently, most clinical-grade instruments use magnetic particles to select specific target cell populations^8,11–13^. These systems offer high quality T cell selection, but the use of directly labeled magnetic particles with high-affinity antibodies targeting cell surface molecules limits the potential of creating a more defined target cell population and may also impact further downstream procedures. For example, already labelled target cells can undergo the selection procedure only once, thus inhibiting opportunities for sequential (positive) selections. To circumvent this and other limitations present in current clinical settings, we have transformed previously described Streptamer-based magnet-free Immuno-Affinity Chromatography (IAC) technology into a GMP-compliant Automated Traceless Cell affinity chromatography (ATC) system intended for clinical applications^9^.

ATC technology enables defined selection of T cell subsets as well as concomitant T cell stimulation in a single closed system suitable for GMP-grade cell manufacturing. This novel automated cell chromatographic purification system is based on the reversible Strep-tag technology platform (Twin-Strep-tag:Strep-Tactin) that utilizes appropriate Fab fragments for column-based parallelized isolation of target cells out of different starting materials (e.g.: leukapheresis). ATC upfront selections include, but are not limited to, isolation of bulk CD3^+^ T cells or parallel co-selection of CD4^+^ and CD8^+^ T cells. Further process improvements include development of on-column T cell manipulations to combine T cell selection and stimulation in an automated fashion. Moreover, we can expand the varieties of the cell source material as well as cell types that can be selected (including more defined T cell subset enrichment). Hence, we believe that ATC is a versatile liquid handling system and an appealing alternative to currently available cell selection techniques used in clinical manufacturing.

## Results

### Components of ATC system (selection resin, tubing set and selection device) enable efficient cell isolation from source material

**Figure 1:**
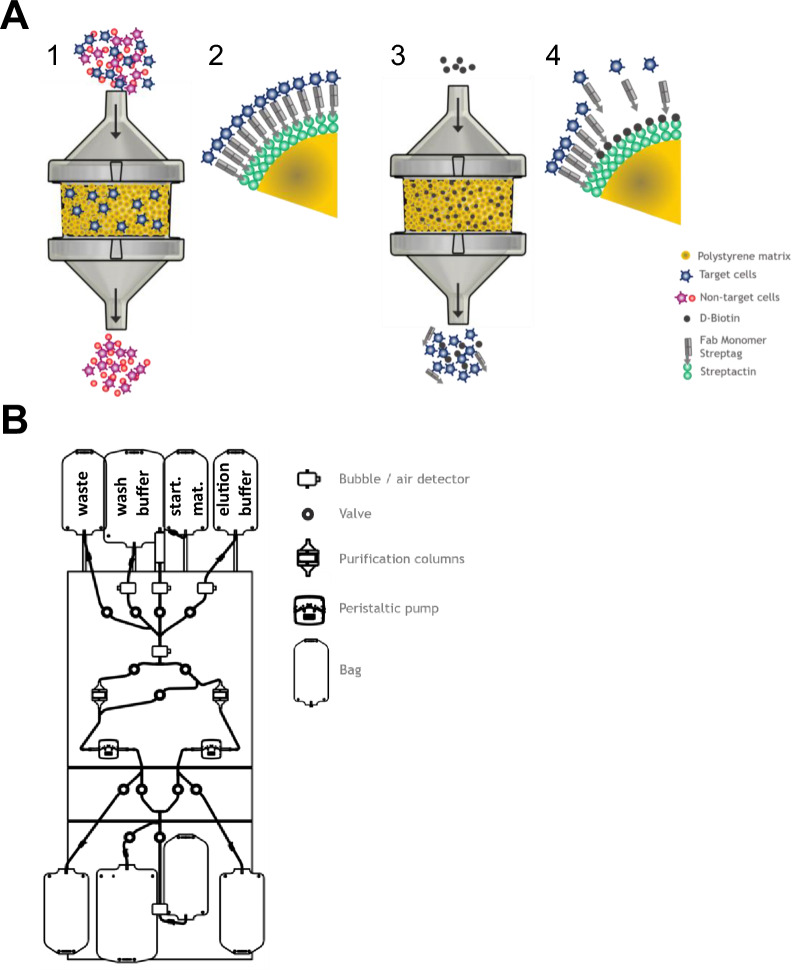
Schematic depiction of automatic column-based selection of target cells using cell affinity chromatography. (**A**) Target cell selection procedure: Starting material is loaded onto the bead-filled column (1) enabling binding of target cells to the specific Fab fragments on the bead surface and flushing out the non-target cells (2). Elution of pure target cells from the column is executed by adding D-biotin buffer (3). Biotin replaces the Fab in the Strep-tag binding pocket and therefore target cells are released and collected (4). (**B**) Schematic drawing of ATC hardware selection device’s front plate with selection tubing set. The device consists of valves for controlling the liquid flow path, peristaltic pumps for transporting liquids, bubble detectors for recording air in the system and column holders with shaking function. The tubing set is a dry, sterile and ready-to-use tubing set for target cell purification and contains two separate columns including pre-mounted empty bags for collecting different cell fractions as well as bags for wash buffer, D-biotin elution buffer and apheresis starting material (as depicted).

ATC cell selection system is a column-based technology that uses previously described reversible Twin-Strep-tag:Strep-Tactin platform^14^. In the ATC system, Strep-Tactin multimer backbone molecules are covalently immobilized on size-defined polystyrene beads (called here selection resin or matrix). Strep-tagged Fab fragments targeting cell-surface molecules of interest are bound to the selection matrix via the Twin-Strep-tag that is C-terminally attached to the heavy chain. This design serves as a reversible target cell binding unit. Cell selection is performed by loading, e.g., a fresh leukapheresis under constant flow onto the column filled with selection matrix (Fig. 1A). Unbound non-target cells pass through the selection resin without binding. After loading the entire starting material, the selection matrix is washed to remove residual trapped non-target cells. The target cell population bound to the selection resin is eluted by flushing the column with D-biotin-containing buffer (Fig. 1A). Due to its high affinity D-biotin efficiently outcompetes Strep-tagged Fab fragments in terms of binding to biotin binding sites on the Strep-Tactin multimer and target cells are rapidly released from the selection matrix (Fig. 1A).

Based on this non-magnetic reversible cell selection technology we concomitantly developed a process, a sterile and ready-to-use tubing set including respective affinity columns and a dedicated fully automated T cell chromatography device (ATC device) for coordinated operations (Fig. 1B). For all three items, we have consulted standard screening and full-factorial DoE (Design of Experiments) approaches. DoE experiments were based on carefully predefined parameters conducted in either small- or full-scale experiments and connected to the specific application (e.g. CD4/CD8 or CD3 positive selection or depletion). The most relevant DoE parameters were found to be liquid flow rates (for starting cell material load, column washing, target cell elution), column geometry, column movement/shaking, purification matrix functionalization (e.g. Strep-Tactin multimer and Fabs), target cell elution profiles, washing and elution buffer composition as well as column temperature for the later versions of the system. An example of optimization-relevant ATC selection parameters required to reach maximal desirability within a given range is presented in Supplementary Fig. 1. Configured ATC device and tubing set allow fast and automatic target cell purification in a fully closed system. Target cell selection studies described in this manuscript, either utilize tubing sets with one target cell adsorbing column or with two columns in series for higher capacities or simultaneous isolation of two different target cell types. Furthermore, we used either full-scale columns (with defined bead matrix sufficient to isolate large target cell numbers appropriate for state-of-the-art cell manufacturing) or small-scale columns representing 1/5th of the full-scale selection matrix. Small-scale purification process was proportionally downscaled for relevant parameters used in the full-scale format using 20% flow rates and a column with 20% cross-section and identical bed height. For each selection type an additional DoE was adjusted depending on individual selection applications and target selection markers by maximizing desirability of individual selection requirements such as purity (Supplementary Fig. 1). Characterization of the entire system was achieved by combining and verifying abovementioned DoE results in full-scale selections.

Full-scale and small-scale tubing sets are dry, sterile, and ready-to-use for target cell purification and are manufactured either in-house or by the tubing set supplier. As depicted in Fig. 1B, the tubing sets are composed of the following components: separate bags for wash buffer, D-biotin elution buffer and cell starting material like washed leukapheresis welded to the top of the tubing set; blood filter/bubble trap directly after the starting material bag; two purification columns (one CD4 and one CD8 column for CD4/CD8 selection process or two CD3 columns for CD3 selection process or columns with other target cell addressing Fab fragments) filled with dry selection matrix at the appropriate scale; bags mounted to the bottom of the tubing set for collecting flow through/waste into one bag and eluted target cell fractions from both columns into two separate bags.

All cell selection studies described here were either performed on clinical-grade pilot-devices or clinical-grade prototypes. All devices were equipped with similar hardware components and controlled by an automation system resulting in identical functionality and consistent data integration. Hardware components relevant for the purification procedure are depicted in Fig. 1B and include valves for controlling liquid flow path, peristaltic pumps for transporting all liquids at a dedicated speed, bubble detectors to control presence of liquid and column holder with shaking function. All ATC affinity resin designs and purification procedures were developed in-house according to the requirements of each specific Fab fragment and target cell of interest. Each purification procedure comprises specific settings for flow speeds, buffer volumes and shaker parameters and takes approx. 90 min (30 min preparation and 60 min hands-off automated procedure) irrespectively of negative or positive cell collection is intended. More detailed descriptions of cell isolations using ATC system are available in “Materials and Methods” section.

### Robust resin manufacturing guarantees reliable target cell isolation

**Figure 2:**
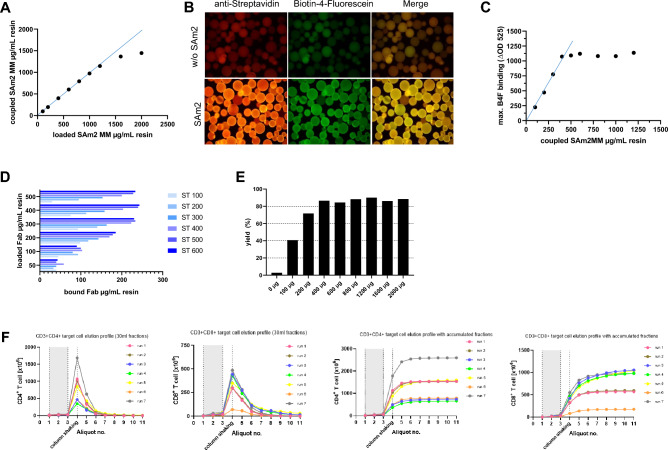
Development and characterization of the selection matrix towards the optimal target cell-capturing performance. (**A**) The linear portion for the immobilization of Strep-Tactin multimer backbone (SAm2 MM) to epoxy functionalized CytoSorbents base matrix is within 100–1000 µg Sam2 MM per mL reconstituted resin. Diagram shows the mean of 5 independent coupling reactions performed in PBS reaction buffer of pH 7.2. (**B**) Surface bound SAm2 MM retains the property to associate with biotin. Pictures show binding of the fluorescent biotin derivate Biotin-4-Fluorescein (FITC) to coupled CytoSorbents raw beads. Coupling reaction was performed in the presence (lower panels) or absence (upper panels) of 500 µg SAm2 MM. SAm2 MM coated surface is visualized by anti-Streptavidin-PE antibody staining. (**C**) The ability of surface bound Strep-Tactin to associate with biotin. The capacity for Strep-Tactin interaction on surface bound SAm2 MM was evaluated by Biotin-4-fluorescein (B4F) quenching assay. Diagram shows the mean of maximal B4F adsorption of three independent experiments. (**D**) Assessment of coupling of Fab fragments to the SAm2 MM. Diagram shows the quantity of Fab fragments bound to 1 mL column matrix dependent on the amount of surface bound SAm2 MM. Quantity of bound Fab fragments was calculated by quantifying residual protein content of Fab coupling solution after 1 h incubation time from the value initially loaded to SAm2 MM-coated matrixes. (**E**) Productivity of 1 mL selection matrix refers to the limit of functional coupleable SAm2 MM. CD3^+^ target cells were purified with increasing amounts of immobilized Fab fragment:SAm2 MM complex at constant ratio. Efficiency of T cell purification was calculated by the ratio of yielded T cell numbers related to the estimated T cell number based on their frequency in starting material. (**F**) Elution profiles of CD4 and CD8 target T cells for collected single fractions (left panels) and accumulated fractions (right panels) for seven independent cell selections performed on the ATC device with different target cell starting counts. Leukapheresis was used as a starting material.

The core of ATC system is a high-quality and ready-to-use selection resin that can be functionalized with specific Fab fragments against cell surface antigen(s). A variety of different custom-made Fab fragments can be functionalized according to the cell type of interest. To provide a GMP-compliant matrix, wet selection resin was adapted from formerly used agarose particles^9^ to dry plastic beads. Generation of the newly developed selection matrix is a multistep manufacturing process based on epoxide chemistry. The epoxide chemistry serves as a stable immobilization method to bind ligands containing amine functional groups, such as Strep-Tactin multimer backbone, whereby the activity of the epoxide functional groups increases with augmenting pH. To evaluate a robust and rapid coupling of Strep-Tactin multimer onto polystyrene beads, we monitored the immobilization of increasing amounts of Strep-Tactin multimer backbone (SAm2 MM) in a time and pH dependent manner. Based on initial findings, we validated a quantitative and reliable coupling of up to 1000 µg SAm2 MM per 1 mL reconstituted raw beads (value below saturation point) in PBS reaction buffer of pH 7.2 within a two-hour reaction time (Fig. 2A). Under these coupling conditions, surface bound Strep-Tactin multimer retains the property to associate with Strep-tagged Fab fragments for target cell binding and D-biotin for target cell elution (Biotin association shown as signal emitted from biotin derivate Biotin-4-Fluorescein) (Fig. 2B).

In order to determine the Fab binding capacity of the resin via specific Strep-Tactin interaction, we quantified the surrogate capability of Biotin-4-Fluorecein (B4F) binding by SAm2 MM coated matrixes in a dose-dependent manner (Fig. 2C). Results from this experiment revealed a significant difference between the total amount and the amount of SAm2 MM that can be functionally immobilized. The linear portion for the quantitative immobilization of SAm2 MM onto base matrix was determined within 100–1000 µg range of SAm2 MM per mL resin (Fig. 2A), whereas the maximum of B4F adsorption is already achieved at about 500 µg SAm2 MM. This finding implicates those quantities of SAm2 MM immobilized onto base matrix exceeding 500 µg per mL resin will not contribute to biotin binding. Since the biotin-specific binding sites of Strep-Tactin are equivalent to the binding sites for Strep-tagged Fab fragments, the same limitation is consequently expected for Strep-tagged Fab fragments (Fig. 2D).

To find the optimal activity of the Fab fragment:SAm2 MM complex on the selection matrix, the ratio of both components has been evaluated by quantifying the binding capacity of immobilized SAm2 MM for Fab fragments (Fig. 2D). As a result, complete binding of 100 µg Fab fragments to 1 mL resin bed volume requires about 200 µg immobilized SAm2 MM which suggests that only 25% of the biotin binding sites on SAm2 MM are bound by Fab assuming that each Fab associated Twin-Strep-tag occupies 2 biotin binding sites. Most probably, this is due to steric limitations. Hence, maximum Fab binding capacity is already achieved by the immobilization of 500–600 µg SAm2 MM suggesting that the active surface capable to bind Fab cannot be increased by the immobilization of SAm2 MM beyond this value (Fig. 2C,D).

To confirm these analytical results on a functional level, Fab related target cells were purified from leukaphereses utilizing increasing amounts of immobilized Fab:SAm2 MM complex at constant 1:2 ratio (Fig. 2E). In line with the evaluated upper limit of Fab binding capacity, maximal yielding of target cells was equally achieved at 500 µg SAm2 MM immobilized per ml resin bed volume confirming that this amount of surface bound Strep-Tactin MM already achieves maximal functionality (Fig. 2E).

To ensure that selected Fab fragment and SAm2 MM amounts bound to the raw resin enable not only efficient capture of target cells but also efficient elution, the D-biotin elution profiles were recorded. Here, D-biotin elution profiles were defined as number of target cells released from selection resin in pre-defined volume increments until complete elution was reached. In this experiment, selection matrix was functionalized with anti-CD4 and anti-CD8 Fab fragments targeting CD4^+^ or CD8^+^ T cells, respectively. Seven independent elution profiles of CD4^+^ T cells as well as for CD8^+^ T cells are depicted in Fig. 2F. The elution behavior was analyzed by splitting the entire elution volume into 11 equal fractions (first 3 fractions were eluted without column shaking, following active shaking for all remaining fractions). The first 3 elution fractions without shaking were showing a very low CD4^+^ and CD8^+^ target T cell count in all seven runs. After starting the shaker at fraction 4, cells elute efficiently and elution was almost complete after collection of fraction 6 (Fig. 2F, right panels).

### ATC system can efficiently select cells from donor starting materials

**Figure 3:**
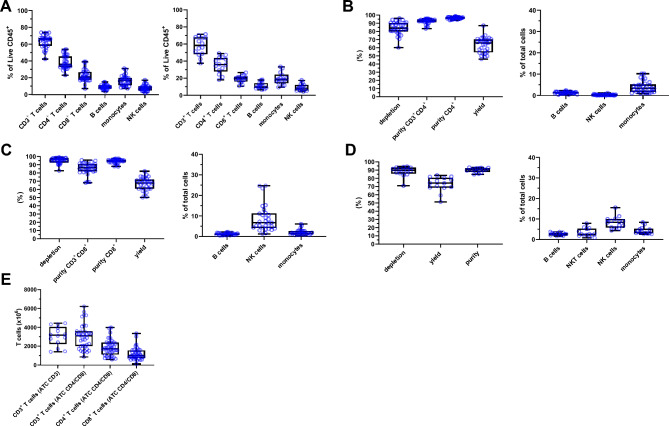
Characterization of ATC T cell purification using leukapheresis as starting material. (**A**) Lymphocyte cell subset composition of starting material. Graphs present data from 27 washed healthy apheresis donors (left panel) and washed cryopreserved apheresis healthy material from 14 donors (right panel). Boxes represent mean ± SD. (**B**) Selection data for CD4 target cell fraction in the ATC CD4/CD8 selection process. Graphs show CD4^+^ T cell depletion efficiency, purity for CD4^+^ T cell target fraction as well as CD4^+^ on-target fraction, CD4 T cell yield and on-target (monocytes) and off-target impurities (B cells, NK cells) of 27 individual purifications. Boxes represent mean ± SD. (**C**) Selection data for CD8 target cell fraction in the ATC CD4/CD8 selection process. Graphs show CD8^+^ T cell depletion efficiency, purity for CD8^+^ T cell target fraction as well as CD8^+^ on-target fraction, CD8^+^ T cell yield and on-target (NK cells) and off-target impurities (B cells, monocytes) of 27 selections. Boxes represent mean ± SD. (**D**) Selection data for CD3 target cell fraction in the ATC CD3 selection process. Graphs show CD3^+^ T cell depletion efficacy, purity for CD3^+^ T cell target fraction, CD3^+^ T cell yield and target fraction impurities (B cells, NK cells, monocytes) of 14 purifications. Boxes represent mean ± SD. (**E**) T cell purification data showing absolute target cell counts from the ATC CD4/CD8 process as well as ATC CD3 process. Graphs represent cumulative data described above. Boxes represent mean ± SD.

Having established optimal selection resin design for anti-CD3, anti-CD4, and anti-CD8 Fab fragments, we tested ATC cell isolation using starting materials commonly used in clinical and commercial T cell manufacturing (Fig. 3). Currently, the most frequent source of immune cells is leukapheresis. It can be processed post-collection in two ways, either fresh or cryopreserved. In Fig. 3A, cell composition spectrum of these two forms is presented (27 fresh and 14 cryopreserved washed healthy donor materials). The fresh leukapheresis collected from healthy European donors had homogenous cell compositions consisting of following median frequencies: 66% CD3^+^ T cells, which broke down into 36% CD4^+^ T cells and 20% CD8^+^ T cells (with remaining 10% accounted to other CD3^+^ cells), as well as 9% CD19^+^ B cells and 17% CD14^+^ monocytes. Cryopreserved leukapheresis materials were obtained from healthy US donors showing median frequencies of 58% CD3^+^ T cells (38% CD4^+^ T cells and 19% CD8^+^ T cells) as well as 10% B cells and 19% monocytes. These values were representative of healthy population peripheral blood mononuclear cell distribution reported in the literature^15^. Of note, higher variation of cryopreserved leukapheresis results most likely from donor pool or apheresis collection rather than from cryopreservation itself as donor comparisons between fresh and cryopreserved material demonstrated little difference.

Utilizing the aforementioned starting materials we evaluated T cell purification efficiency of ATC CD4 (Fig. 3B), CD8 (Fig. 3C) as well as CD3 selections (Fig. 3D) by assessment of three basic parameters: depletion (difference between absolute number of target cell population present in starting material and flow through after selection divided by the absolute number of target cells in starting material), (T cell) purity, and yield (absolute number of target cells isolated and eluted compared to total number of target cells in starting material). Across the ATC CD4/CD8 co-selection processes CD3^+^CD4^+^ (T) cell depletion efficiencies were consistently above 60% with a median of 82% (Fig. 3B). Similar, the CD3^+^CD8^+^ (T) cell depletions were constantly above 70% with a median of 95% (Fig. 3C). Eluted fraction purities consistently reached values above 80% for CD3^+^CD4^+^ T cells with a median of 93% in CD4^+^ fractions (Fig. 3B) and around 70% with a median of 86% for CD3^+^CD8^+^ T cells in the CD8^+^ fractions (Fig. 3C). Combining selected on-target T cells and other on-target subsets the overall median purity for CD4^+^ was at 97% (Fig. 3B) and for CD8^+^ at 95% (Fig. 3C). In addition, T cell yields were at 66% for CD4^+^ T cells (Fig. 3B) and at 67% for CD8^+^ T cells (Fig. 3C). For CD3 selections, the target cell depletions were constantly over 70% with a median of 90%, target cell purities were at least 85% with a median of 91% and a median yield was calculated at 74% (Fig. 3D). Noteworthy, two purification full-scale columns have fixed maximum target cell capture capacity estimated at around 4000 × 10^6^ total target cells. Therefore, very high starting T cell numbers above this value may result in lower relative yields.

In regard of on-target impurities, we observed expectantly highest number of monocytes post-isolation in CD4 selections (due to presence of CD4 marker on some of monocyte subspecies) and NK cells in CD8 selections (because of CD8 marker expression by NK subpopulations) (Fig. 3B–D). In case of off-target contaminations, very low levels of other cell types were found in all purifications (< 3%) indicating very high specificity of ATC selections (Fig. 3B–D). Moreover, to rule out a potential bias in enriching certain T cell subsets when using ATC selection, T cell subset composition of starting and selected materials were compared based on expression profiles of typical T cell surface markers (characterized surface markers are listed in “Materials and Methods”) and showed no difference in phenotypic subset composition prior to and following ATC selection. For clarity we used an example of CD3 selection as CD3 antigen is a pan T cell marker (Supplementary Fig. 2). First, we detected no skew in CD4:CD8 T cell ratio during selection (Supplementary Fig. 2A). Second, we observed identical phenotypic T cell composition in starting and selected materials (Supplementary Fig. 2B,C). Third, apparent variations in T cell subset composition were driven by the variability among donors as indicated by Principal Component Analysis of T cell phenotypes (Supplementary Fig. 2C). Therefore, the ATC system enables highly specific and unbiased target cell isolation.

Most importantly, the ATC CD4/CD8 co-selection as well as CD3 purifications constantly delivered more than 1000 × 10^6^ total T cells per selection with a median of approx. 3000 × 10^6^ cells (Fig. 3E). Moreover, CD4 and CD8 isolations yielded median of 2000 × 10^6^ and 1000 × 10^6^ cells, respectively (Fig. 3E). Importantly, ATC selection appeared to be as efficient with more challenging cell compositions that represent more closely patient material (i.e., high monocyte and B cell content but low T cell frequency) (Supplementary Fig. 3). In summary, ATC purification is suitable for isolation of large numbers of highly pure T cell subsets from conventionally used cell source material.

### Custom-made Fab binders extend applicability of ATC beyond classical upfront T cell selection

As described above, ATC technology is very suitable for T cell isolation from leukapheresis based on CD3, CD4 and CD8 markers. However, the flexibility of this technology enables even more precise enrichment of certain T cell subset targeting phenotype-specific antigens. It has been proposed that phenotype-based T cell selection may have a substantial benefit on immunotherapy outcome and T cells expressing markers such as CD62L, CCR7, CD27 and/or CD28 were recognized as potential isolation targets^16^.

**Figure 4:**
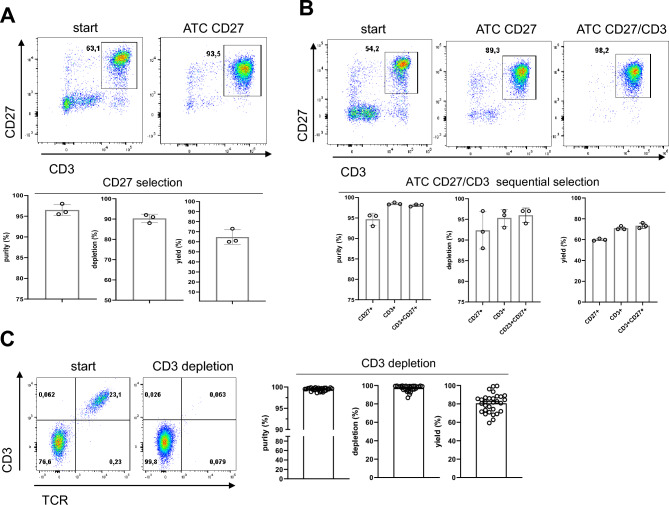
Examples of multiplexed ATC selections for the generation of more homogenous T cell populations. (**A**) ATC CD27 selection efficiently enriches for CD27^+^ cells. Dot plots of one representative selection are shown. Graphs display purity, depletion, and yield based on the frequency of CD27^+^ cells. Cells were pre-gated on live, single CD45^+^ lymphocytes. Graph summarizes data from 4 independent measurements. Bars represent mean ± SD. (**B**) ATC CD27/CD3 selection isolated highly pure CD3^+^CD27^+^ T cells. Dot plots of one representative selection are shown. Graph shows purity, depletion, and yield of either ATC CD27, ATC CD3 or ATC CD27/CD3 selections. Cells were pre-gated on live, single CD45^+^ lymphocytes. Graph summarizes data from 3 independent measurements. Bars represent mean ± SD. (**C**) ATC CD3 selection can be utilized to remove CD3^+^ non-edited cells from allogenic T cell product. ATC CD4/CD8 pre-selected T cells were stimulated with Expamers and after 48 h gene edited to knock-out TCR/CD3 expression. Upon the cell expansion, ATC CD3 depletion was performed. Representative dot plots of the flow cytometry staining are shown. Cells were pre-gated on live, single CD45^+^ lymphocytes. Graphs summarize data from 30 independent measurements. Bars represent mean ± SD.

One example of enhancing T cell isolation is the use of CD27 surface marker as a positive selection step. CD27 is expressed on all subsets of T cells tending to be stronger upregulated in younger and healthier donors^17^. Most importantly, higher content of CD27^+^ T cells was correlated with better outcome of immunotherapy in treated patients^18^. Therefore, evaluating the potential of ATC CD27 purification is of high relevance (Fig. 4A). CD27 positive selection on the ATC resulted in purity of 95%, depletion of 90% and yield of 65% confirming potential application (Fig. 4A). However, CD27 positive selection requires D-biotin-mediated elution step preceding any further downstream T cell isolation. As a consequence, an in-process washing strategy was developed for removal of D-biotin and Fab fragments from the selected material to allow multiple successive positive selections. To study the efficiency of such sequential selection, we first performed the CD27 positive selection followed by CD3 positive selection (Fig. 4B). As a result, we were able to isolate CD27^+^CD3^+^ double-positive T cells to a final purity of above 95% maintaining high depletions and yields for both selections (Fig. 4B).

Additionally, we evaluated the potential expansion of ATC selection beyond T cells (Supplementary Fig. 4). We were interested in addressing other peripheral blood mononuclear cell lineages. Hence, we tested monocyte and NK cell isolation using Fab fragments targeting CD14^+^ and CD56^+^, respectively (Supplementary Fig. 4A and 4B). We were able to show that ATC selection with these markers is feasible as well. Noteworthy, CD14^+^ and CD56^+^ (and others like CD19^+^) Fab fragments can be of use in multiple-selection setups aiming at yielding ultra-pure T cells.

Further, we considered application of ATC selections at later stages of cell manufacturing processes. For example, in allogeneic T cell production an additional depletion step after gene editing and before the final cell harvest is necessary. In allogeneic settings, it is critical to remove non-edited and potentially alloreactive T cells expressing native TCR from the final product to prevent TCR-mediated graftversus host disease (GvHD) in treated recipients^19–21^. One approach to decreasing potential alloreactivity includes disruption of the T cell receptor alpha constant (TRAC) locus through precise editing with CRISPR/Cas systems, which inhibits TCR complex formation and trafficking of CD3 to the cell surface^22^. Due to commonly observed incomplete TCR knock-out in gene-edited T cell populations and consequent presence of the remaining non-edited TCR^+^CD3^+^ cells, allogeneic products can also require an additional purification step to reach ultra-high purities^21^. For this purpose, unwanted TCR^+^CD3^+^ cells were depleted after T cell culture from allogeneic cell product by ATC CD3 negative selection (Fig. 4C). As a result, highly pure final allogeneic T cell product was collected as flow-through fraction, reaching desired purities of > 99% for TCR^-^CD3^-^ T cells (Fig. 4C).

Finally, ATC purification can be utilized to generate highly pure Chimeric Antigen Receptor (CAR) T cell products by removing non-edited CAR^-^ T cells. Here, we decided to focus on a truncated human epidermal growth factor receptor polypeptide (EGFRt) that has, in some cases, been co-expressed on the surface of CAR^+^ T cells and serves as safety mechanism by enabling the use of in vivo cetuximab-mediated cell ablation^23,24^. By capturing expanded CAR^+^ T cells via inert EGFRt receptor, we were able to efficiently enrich for CAR transgene and generate pure CAR T cell population (Supplementary Fig. 4C). Taken these data together, we were able to exemplify the versatility of ATC selection system.

### Magnet-free system broadens ATC applications beyond cell selection

**Figure 5:**
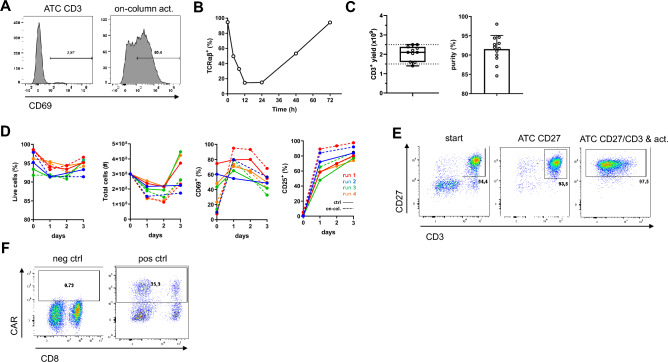
Integration and automation of ATC system with other manufacturing-relevant operations. (**A**) On-column stimulation with Expamers leads to T cell activation. Target cells captured on CD3 selection matrix were left unstimulated or stimulated by addition of Expamers and culture media to the selection column and incubation at 37 °C for 4 h. Collected cells were analyzed using flow cytometry. Cells were pre-gated on live, single CD45^+^ lymphocytes. Histograms depicting expression of CD69 activation marker from one out of 12 experiments are shown. (**B**) TCR/CD3 complex is downregulated upon activation. Graph shows TCR expression kinetics up to 72 h after an ATC CD3 selection with on-column activation. Graph represents data from one experiment. (**C**) A predictable number of target cells can be captured under saturating conditions. Graphs show CD3^+^ cell yield and purity of several ATC CD3 single column selections. Graph summarizes data from 11 independent measurements. Bars and boxes represent mean ± SD. (**D**) Comparison between ATC CD3 selection with either simultaneous or subsequent T cell stimulation. Kinetic of viability, cell proliferation and expression of early and late activation markers. Collected cells were counted and analyzed using flow cytometry. Cells were pre-gated on live, single CD45^+^ lymphocytes. Graphs show cumulative data from 4 experiments. (**E**) On-column activation of CD3^+^CD27^+^ T cells. CD3^+^CD27^+^ T cells were isolated using ATC CD27/CD3 double positive selection followed by on-column stimulation. Representative dot plots are shown. (**F**) Example of on-column activation and downstream transduction. Selected, stimulated and subsequently transduced (pos ctrl) or not (neg ctrl) T cells were analyzed for CAR transgene expression after 5 days of culture using flow cytometry. Cells were pre-gated on live, single CD45^+^ lymphocytes. Dot plots depicting expression of CAR receptor from one out of 3 independent experiments are shown.

Lack of the necessity to use magnetic particles and non-removable high-affinity antibodies during selections opens new use opportunities for ATC. To this end, we investigated whether ATC can integrate and automate additional operations relevant for cell manufacturing (Fig. 5). First, we assessed if T cell stimulation can be induced already during above-described ATC CD3 selection (while the T cells are directly attached onto the selection matrix) instead of doing this post selection in a separate step. For this purpose, we used another Streptamer-based GMP-compliant technology: the anti-CD3/anti-CD28 activation reagent called Expamers^25^. Expamers are proteinaceous soluble particles that can be readily introduced into ATC liquid handling system. Addition of Expamers directly onto CD3^+^ T cells captured on selection column followed by short incubation at 37 °C led to cell activation demonstrated by rapid expression of canonical T cell activation marker CD69 (Fig. 5A).

Surprisingly, after approx. 4 h of on-column incubation, T cells started to spontaneously detach from selection matrix. To understand observed Activation-Induced Detachment (AID), we studied TCR expression dynamics upon stimulation. It has been previously reported that anti-CD3/anti-CD28-mediated stimulation results in fast downregulation/internalization of CD3/TCR complex from the T cell surface^22,26^. Indeed, direct measurement of TCR presence (using anti-TCRαβ antibody to avoid interference from CD3 Fab fragments) revealed downregulation of the receptor within hours after on-column activation (Fig. 5B). Therefore, already after 4 h of on-column incubation together with Expamers, the expression of the CD3 receptor decreased sufficiently and allowed release of the activated cells without the need to add D-biotin. To better control the strength of activation signal induction, we decided to use one column only for CD3 selection in this application. Knowing maximum capacity of single column (under saturating conditions provided by average-sized leukapheresis), we expected reliable binding of 2000 ± 500 × 10^6^ of target T cells to which we can add defined amount of Expamers. Limiting absolute T cell numbers within expected range by utilization of single column was shown in 12 independent measurements (Fig. 5C). Within these settings we tested temperature ranges and extended on-column incubation times to optimally determine activation procedure on the ATC (Supplementary Fig. 5). Of note, inclusion of on-column activation extends time of ATC program to approx. 5 h but this is a fully automated procedure requiring similar to ATC standard selection only 30 + 30 min hands-on operations pre- and post-selection.

In an effort to compare the performance of on-column activated and AID-released T cells to cells activated after D-biotin elution, we performed head-to-head comparison experiments of downstream cultures (Fig. 5D). ATC-isolated T cells activated either simultaneously or successively were cultured for 3 days in media supplemented with cytokines. We observed a high viability throughout both cultures but increased total cell numbers at harvest for on-column activated T cells compared to T cells activated after D-biotin elution (Fig. 5D). Furthermore, immediately after the AID, the cells showed an increased expression of the early activation surface marker CD69, which decreased over time. However, T cells from both activation methods had comparable expression patterns for the late activation surface marker CD25 with a high presence up to day three (Fig. 5D).

To examine feasibility of combining sequential selection and on-column activation, we performed the ATC CD27 selection with consequent ATC CD3 selection that included on-column activation (Fig. 5E). As anticipated, we were able not only to enrich for CD3^+^CD27^+^ cells but also activate them in the process (Fig. 5E). Finally having CAR T cell manufacturing in mind, we tested whether synergistic selection and activation has no negative impact on downstream operations most importantly CAR transduction. As presented in Fig. 5F, on-column activation led to efficient transgene integration and CAR expression within expected ranges after 5 days of cell culture. In conclusion, ATC column selection expanded potential applications of ATC system enabling integration and automation of several separate manufacturing operations such as cell stimulation and genetic modifications.

## Discussion

In this manuscript an affinity chromatographic cell selection technology called ATC intended for clinical (T) cell manufacturing is presented. ATC represents the culmination of Strep-tag technology progression that started 30 years ago.

Strep-tag technology was initially developed for the purification of weakly associated antibody Fv fragments as intact heterodimeric complexes using the tag at the heavy chain^27^. The physiological buffer conditions used throughout the whole purification process in combination with D-biotin mediated gentle elution enabled the functional isolation of higher order membrane protein complexes^28,29^. Because of these initial results, Strep-tag technology rapidly gained popularity particularly for the functional isolation of labile proteins^30^. With the development of Streptamer technology in the early 2000s exploiting the Strep-tag with low affinity MHCs in a novel innovative setting the antigen specific isolation of minimally manipulated human T cells (without any affinity reagents remaining on the cell surface) became possible for the first time^14^. Streptamer technology was then expanded to any cell type by switching from MHCs to engineered low affinity Fab fragments that can be directed to virtually any cell surface molecule^31^. Reversible Streptamer technology used on magnetic nanoparticles was a fundamental progress as compared to the state-of-the-art procedure consisting of irreversibly labelling the target cells with murine monoclonal antibody coated nanoparticles^32^. As a result, Streptamers made isolation of pure and immunogen free cells exhibiting authentic physiologic properties available for therapeutic applications.

A further evolution was the translation of Streptamer-technology to magnet free, column cell immuno-affinity chromatography^9^ that finally led to a closed, fully integrated, GMP-compliant ready-to-use cell purification system described here. ATC uses a more large-scale manufacturing-friendly raw resin and a Fab fragment generation platform. In addition, it is a modular technology which permits combination of separate unit operation beyond cell isolation (e.g., stimulation). The ATC platform offers the possibility to achieve high throughput enrichment of desired cells from various starting materials with a seamless integration into upstream and downstream cell manufacturing processes.

To achieve this, we developed a proprietary liquid handling unit as well as sterile single use tubing sets customized for each type of selection. All selections are controlled by manufacturing grade automation to facilitate scaling out and integrating with other hardware elements required for commercial scale pharmaceutical cell production. Moreover, we ensured that incremental modifications, such as introduction of a heating element necessary for on-column activation operations, are backward-compatible and maintain one-fits-all setup. However, conversely to other systems production of tubing sets with pre-configured Fab ligand density extends lead time for ligand titration for new applications and has to be taken into consideration when designing a new cell manufacturing program. Using described GMP-grade hardware, we demonstrated that ATC technology is appropriate for large volume T cell selection for a broad range of classical selection ligands, such as anti-CD3, anti-CD4, and anti-CD8, widely used in manufacturing processes that require reliable median target cell yields of approx. 3000 × 10^6^ cells with high on-target purities. Besides the common use of leukapheresis products as starting cell source, ATC will be compliant with industry requirements to access purified target cells from other logistically and medically more beneficial sources. The opportunity to enrich cells directly from new sources can potentially simplify manufacturing logistics, especially when focusing on integrated automated manufacturing procedures aiming at generation of differentiated cell products including minimized cell manipulation and total process duration times. Furthermore, aforementioned short and automated selection procedure has a potential to improve the throughput of cell processing at manufacturing site, where sufficient number of ATC units is introduced.

The ATC system expands its applications beyond classical T cell selections. In this manuscript, we have presented how it can be used for a more complex and integrated manufacturing operations to enable next generation cell products. These comprise generation of differentiated cell products like, e.g., more precisely defined homogenous T cell populations or other cell types but also include process associated improvements ranging from downstream polishing applications (such as removal of senescent or non-edited cells) to neatness integration of subsequent processing steps. We also presented a selection framework that can be quickly adapted to any given needs including generation of binders against low affinity antigens as previously shown with the selection using MHC class I molecule^9^.

Finally, we showed that the ATC system can be extended by integration and automation of additional separate operations performed during cell manufacturing. Chromatographic cell purification technology and instrumentation in combination with next generation bioengineering approaches provide an attractive integrated platform. The platform enables a fully closed system with a higher degree of automation e.g., by unified on-column operations such as selection, Expamers activation and potentially genetic modification. This offers the opportunity to transform the current central manufacturing model towards a more decentralized approach that could retain broader cell properties, improve cell production turn-around times, minimize hands-on failures, and ultimately reduce manufacturing costs for cell therapies.

## Materials and methods

### Blood samples

Leukapheresis products of healthy donors’ and preselected donors﻿’ material were collected at CMS Cellex Medical Services GmbH under the ethical quote (Ethical committee of the Technical University Dresden: EK309072016). Usage of the blood samples was approved according to national law by the local Institutional Review Board and the declaration of Helsinki and Istanbul (Ethics committee of the Faculty of Medicine, Technical University of Munich: 360/13 and 55/14). Informed consent was obtained from all subjects and/or their legal guardian(s).

Preselected donor material for multiplexed selections was specifically chosen by the low expression of CD27. To determine marker expression, blood samples were stained with antibodies against CD27 (BV605; clone O323; Biolegend).

Leukapheresis was washed by using Sepax S-100 fully automated cell separation system (Cytiva) with FACS buffer containing 1 × PBS (Thermo Fischer Scientific) with 0.5% HSA (CSL Behring).

### Selection resins characterization

Strep-Tactin multimer backbone (SAm2 MM) as well as Fab fragments targeting specific cell surface antigens were generated according to in-house SOPs as previously described (Poltorak et al., 2020). Selection resin manufacturing was performed according to in-house SOP as well.

To quantify SAm2 MM coupling efficiency, phosphate reaction buffer of pH 7.2 (GE Healthcare) was supplemented with increasing amounts of SAm2 MM and immobilization to raw beads was monitored by taking aliquots of coupling solutions at defined time points. Protein content of aliquots was quantified by colorimetric DC protein assay (Bio-Rad), following the manufacture﻿’s instructions. The amount of coupled SAm2 MM was calculated by deducting residual protein content of aliquots from the initial protein content of the appropriate SAm2 MM coupling solution. To perform fluorescence staining of coated column-matrix, coupling reaction was done in the presence or absence of 500 µg SAm2 MM per mL resin. Coupled beads were stained with anti-Streptavidin-PE antibody (BioLegend). Stained resin was washed and co-stained with the fluorescent biotin derivate Biotin-4-Fluorescein (Sigma). Imaging of stained resins was performed with Biozero fluorescence microscope (Keyence). The binding capacity of SAm2 MM-coated matrices was assessed by B4F quenching assay, as described elsewhere^33^. The quenched signal of B4F at 525 nm is associated with the specific adsorption of this molecule by streptavidin interaction, which is equivalent to the binding site for Strep-tagged Fab fragments on Strep-Tactin. B4F (Sigma) fluorescence intensity of each titration step was deducted from the appropriate emission values of an uncoated resin blank control. The maximal value of blank-corrected signal indicates the saturation of binding sites for Strep-tagged Fab fragments on SAm2 MM-coated matrices. To evaluate the Fab-to-SAm2 MM ratios, epoxy-functionalized raw beads (CytoSorbents) were coated with the indicated amounts of SAm2 MM. SAm2 MM coated matrices were washed and resuspended in Fab-coupling solutions with indicated Fab content. After one hour reaction time, the protein content of Fab coupling solutions was quantified by the colorimetric DC protein assay (Bio-Rad), following the manufacturer’s instructions. The amount of coupled Fab fragments was quantified by deducting residual protein content of supernatants from the initial content of the corresponding Fab coupling solution. SAm2-Fab fragment complex cell binding capacity and elution profiles were performed similar to other cell selections as described below.

### Cell selection using ATC

All tubing sets used in these studies containing dry and sterile selection resins were designed in-house and manufactured either by Raumedic AG or in-house.

All cell selection procedures (unless indicated otherwise) were executed as follows: the “wash buffer bag”, “elution buffer bag” and “starting material bag” were filled with appropriate solutions (wash buffer: 1 × PBS from Thermo Fischer Scientific with 0.5% HSA from CSL Behring; elution buffer: wash buffer containing 0.1–1 mM of D-biotin (produced in-house)) and welded to the dry, sterile tubing set using a sterile tubing welder (Terumo BCT). Afterwards, the tubing set was installed to the ATC device according to the in-house SOP. Directly after tubing set installation, the cell purification process was started and executed automatically. Briefly, process starts with line priming into all bags to remove air followed by rehydration and washing of the initially dry selection matrix in columns. Subsequently, the starting material is loaded onto the column(s). After loading, unbound cells are washed out from column(s) into bag(s) collecting the flow-through/negative fraction. The subsequently performed elution of bound cells with D-biotin buffer from column(s) into positive fraction bag(s) completes the cell purification procedure. For sequential selections, isolated cells were washed to remove D-biotin and Fab fragments. Washed cells were loaded as the input for subsequent further selection steps.

In case of Activation-Induced Detachment (AID), D-biotin buffer was replaced with activation buffer (Expamers diluted in serum-free media containing IL-2, IL-7, and IL-15 cytokines (Poltorak et al., 2020)) and after loading step complete column void volume was exchanged to activation buffer. Next, column bound target cells and activation buffer was warmed up to 37 °C for 4.5 h. Afterwards, cells were eluted into collection bag by flushing column with additional activation buffer.

### T cell phenotyping

All lymphocytes, pre- and post-selection, were analyzed using flow cytometry and the following antibodies: anti-CD45 (AF700; clone HI300), anti-CD3 (PE, FITC or PE-Cy7; clone OKT3), anti-CD8 (PE or BV510; clone HIT8a), anti-CD4 (BV421 or BV785; clone OKT4), anti-CD27 (BV785; clone 323), anti-CD14 (PC7; clone HCD14), anti-CD56 (APC; clone MEM-188), anti-EGFR (PE; clone AY13), TCRαβ (APC-Fire750; clone IP26) and anti-CD19 (BV421; clone HIB19) (all from Biolegend). To determine T cell activation state, T cells were stained with CD69 antibody (APC-Fire750; clone FN50) and CD25 antibody (BV650; clone BC96) (Biolegend) at indicated time points after initial stimulation. For live/dead discrimination propidium iodide (Sigma) was used. CAR expression has been monitored using an anti-idiotype antibody specific for the CD19-directed CAR, an in-house developed antibody. Cell associated fluorescence was analyzed by flow cytometry using either CytoFLEX or CytoFLEX LX flow cytometers (Beckman Coulter), unless indicated otherwise.

Live nucleated cells were enumerated pre- and post-selection (including fractions and downstream culture) using either NucleoCounter® NC-3000™ (ChemoMetec) or XN-350 Cell Counter (Sysmex) according to manufacturers’ instructions.

### T cell activation, gene-editing and culture

T cell stimulation, gene-editing and downstream culture was performed as previously described (Poltorak et al., 2020).

### Data analysis and statistics

Flow cytometric data were analyzed using FlowJo software (FlowJo, LLC). Graphs and statistical analysis were generated using GraphPad Prism software (GraphPad Software).

### Supplementary Information

Supplementary Information.

## Data Availability

The authors declare that all data generated or analyzed for this study are available within the paper and its supplementary information. Additional raw data are available from the corresponding author upon reasonable request. ATC is a proprietary technology of Bristol-Myers Squibb Company and some information are considered trade secret. Specific information about ATC technology can be made available upon reasonable request.
