# Supplementary material for: Next generation automated traceless cell chromatography platform for GMP-compliant cell isolation and activation
Source: Sci Rep. 2022 Apr 21;12:6572. doi: 10.1038/s41598-022-10320-x (PMC9023455; doi:10.1038/s41598-022-10320-x)
Supplement: Supplementary file 1 — Supplementary Information. [file 41598_2022_10320_MOESM1_ESM.pptx]

## Slide 1
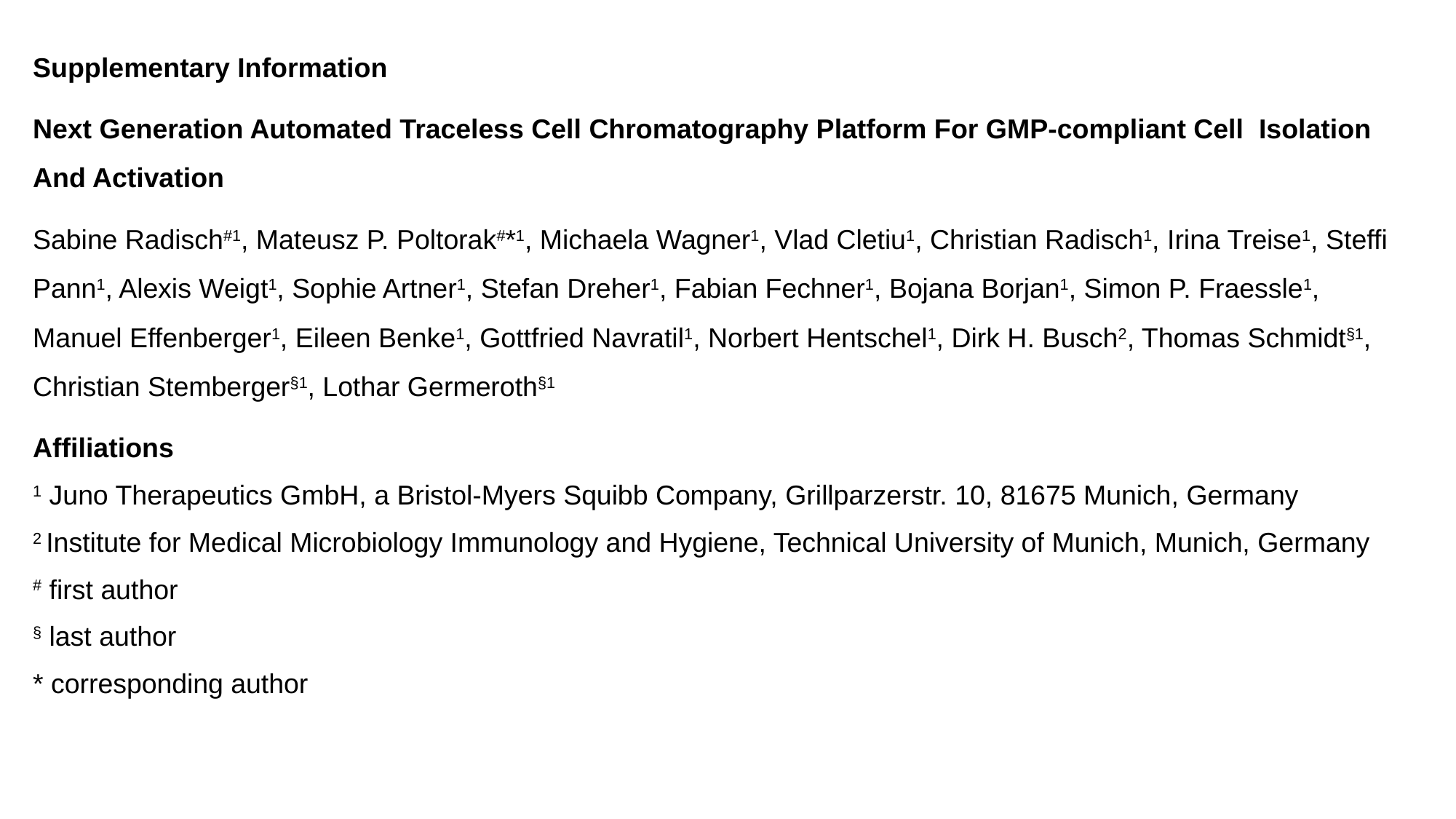

Supplementary Information
Next Generation Automated Traceless Cell Chromatography Platform For GMP‑compliant Cell Isolation And Activation
Sabine Radisch#1, Mateusz P. Poltorak#*1, Michaela Wagner1, Vlad Cletiu1, Christian Radisch1, Irina Treise1, Steffi Pann1, Alexis Weigt1, Sophie Artner1, Stefan Dreher1, Fabian Fechner1, Bojana Borjan1, Simon P. Fraessle1, Manuel Effenberger1, Eileen Benke1, Gottfried Navratil1, Norbert Hentschel1, Dirk H. Busch2, Thomas Schmidt§1, Christian Stemberger§1, Lothar Germeroth§1
Affiliations
1 Juno Therapeutics GmbH, a Bristol-Myers Squibb Company, Grillparzerstr. 10, 81675 Munich, Germany
2 Institute for Medical Microbiology Immunology and Hygiene, Technical University of Munich, Munich, Germany
# first author
§ last author
* corresponding author

## Slide 2
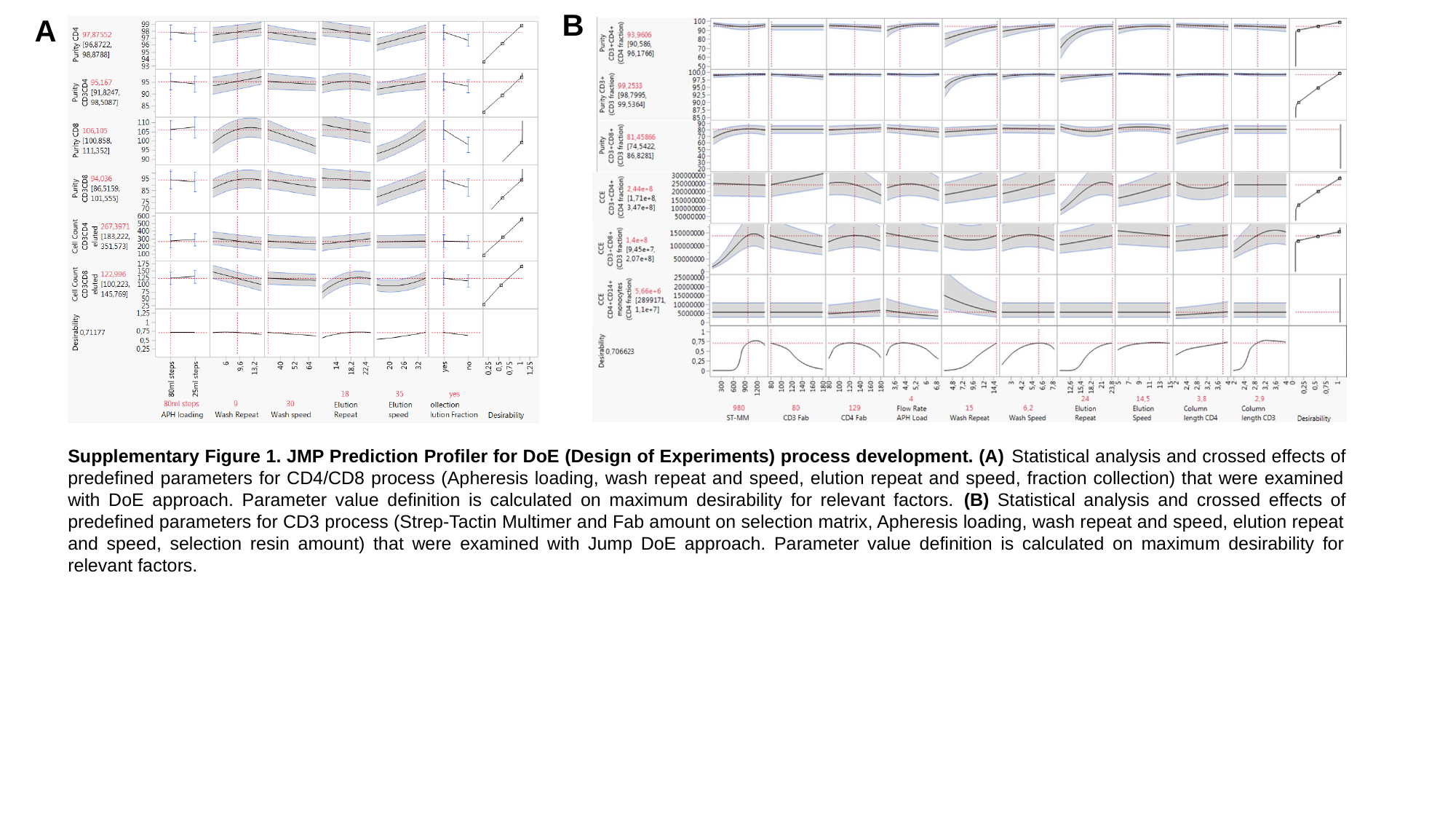

B
A
Supplementary Figure 1. JMP Prediction Profiler for DoE (Design of Experiments) process development. (A) Statistical analysis and crossed effects of predefined parameters for CD4/CD8 process (Apheresis loading, wash repeat and speed, elution repeat and speed, fraction collection) that were examined with DoE approach. Parameter value definition is calculated on maximum desirability for relevant factors. (B) Statistical analysis and crossed effects of predefined parameters for CD3 process (Strep-Tactin Multimer and Fab amount on selection matrix, Apheresis loading, wash repeat and speed, elution repeat and speed, selection resin amount) that were examined with Jump DoE approach. Parameter value definition is calculated on maximum desirability for relevant factors.

## Slide 3
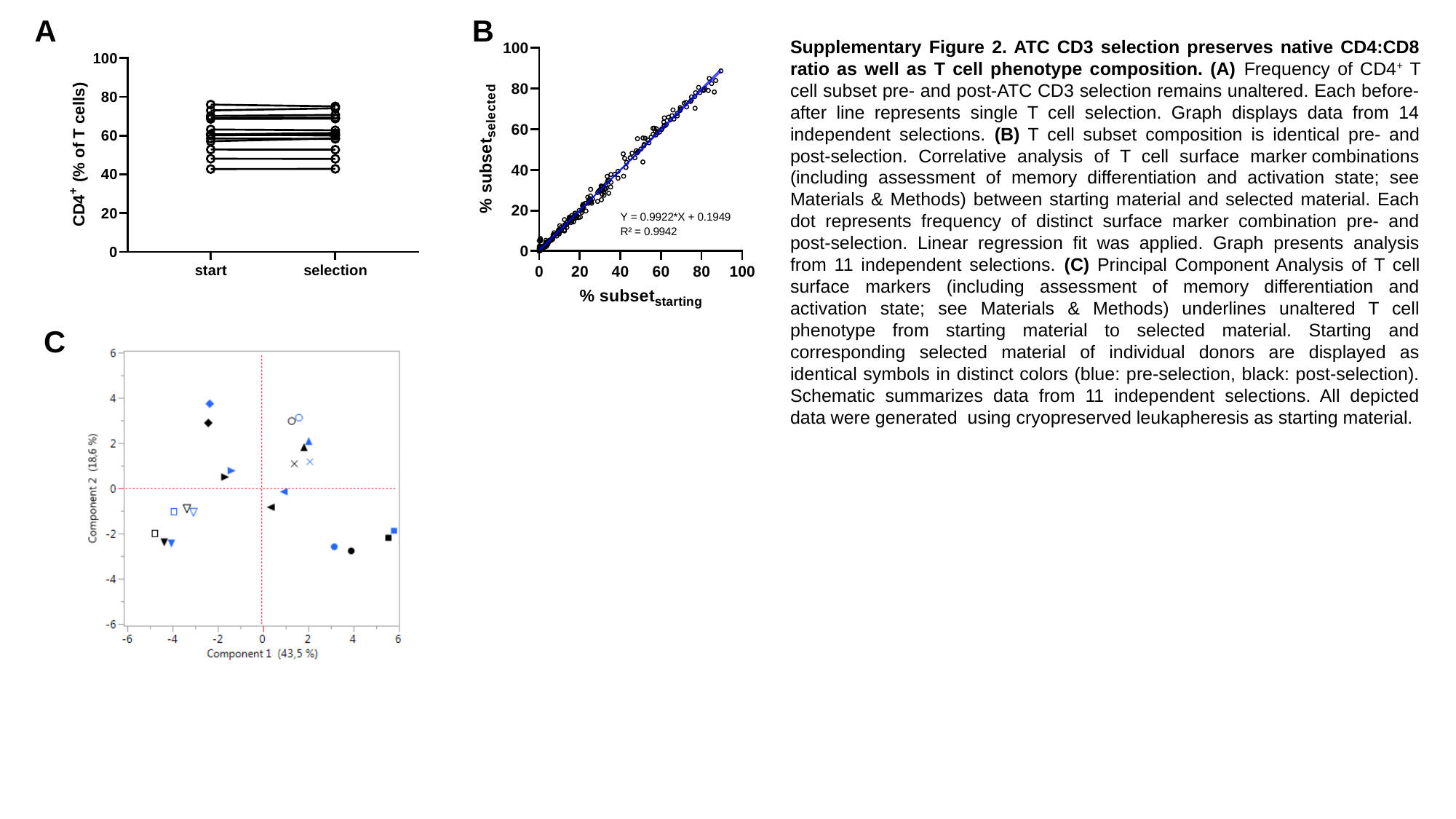

A
B
Supplementary Figure 2. ATC CD3 selection preserves native CD4:CD8 ratio as well as T cell phenotype composition. (A) Frequency of CD4+ T cell subset pre- and post-ATC CD3 selection remains unaltered. Each before-after line represents single T cell selection. Graph displays data from 14 independent selections. (B) T cell subset composition is identical pre- and post-selection. Correlative analysis of T cell surface marker combinations (including assessment of memory differentiation and activation state; see Materials & Methods) between starting material and selected material. Each dot represents frequency of distinct surface marker combination pre- and post-selection. Linear regression fit was applied. Graph presents analysis from 11 independent selections. (C) Principal Component Analysis of T cell surface markers (including assessment of memory differentiation and activation state; see Materials & Methods) underlines unaltered T cell phenotype from starting material to selected material. Starting and corresponding selected material of individual donors are displayed as identical symbols in distinct colors (blue: pre-selection, black: post-selection). Schematic summarizes data from 11 independent selections. All depicted data were generated  using cryopreserved leukapheresis as starting material.
C

## Slide 4
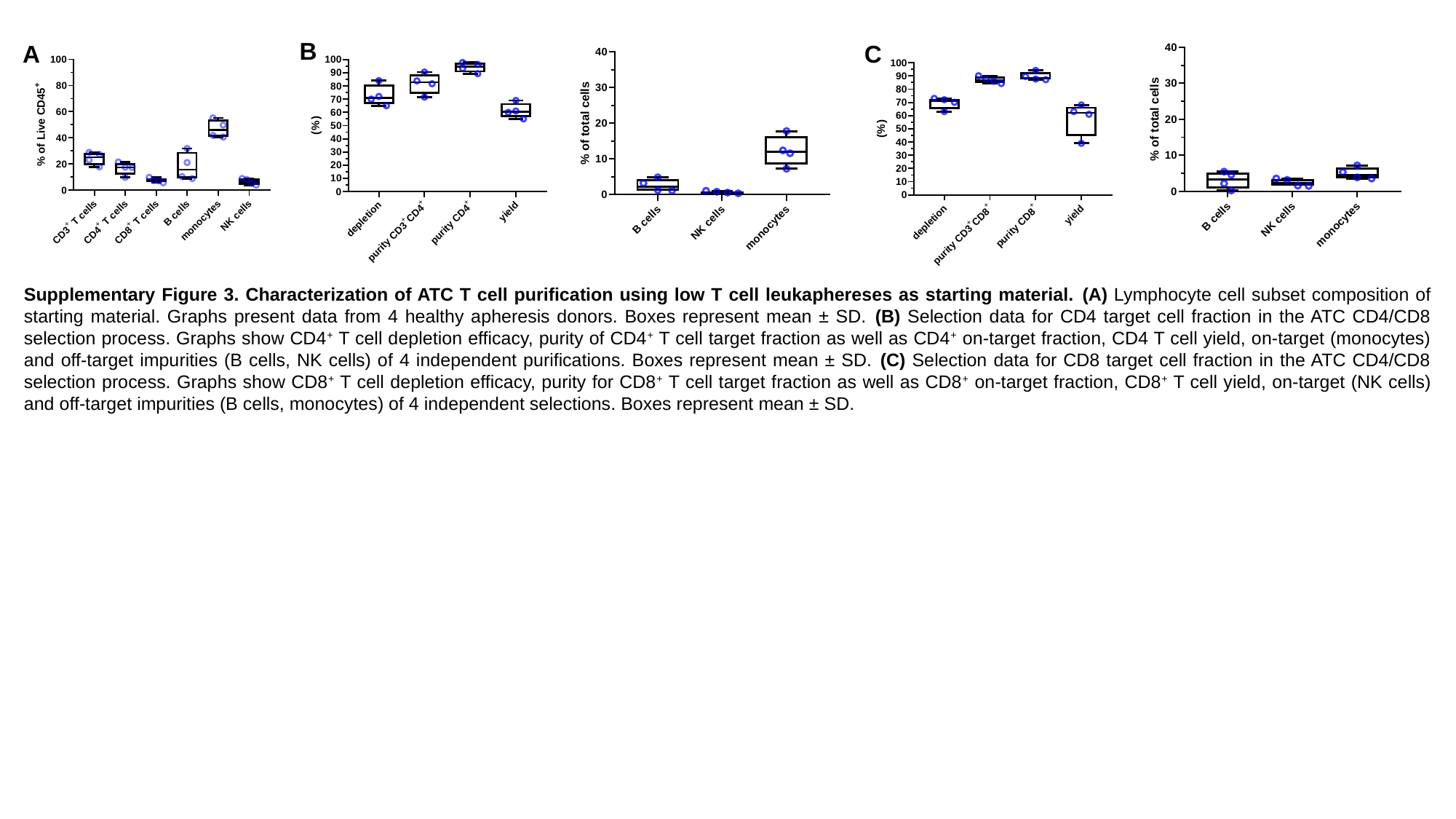

B
A
C
Supplementary Figure 3. Characterization of ATC T cell purification using low T cell leukaphereses as starting material. (A) Lymphocyte cell subset composition of starting material. Graphs present data from 4 healthy apheresis donors. Boxes represent mean ± SD. (B) Selection data for CD4 target cell fraction in the ATC CD4/CD8 selection process. Graphs show CD4+ T cell depletion efficacy, purity of CD4+ T cell target fraction as well as CD4+ on-target fraction, CD4 T cell yield, on-target (monocytes) and off-target impurities (B cells, NK cells) of 4 independent purifications. Boxes represent mean ± SD. (C) Selection data for CD8 target cell fraction in the ATC CD4/CD8 selection process. Graphs show CD8+ T cell depletion efficacy, purity for CD8+ T cell target fraction as well as CD8+ on-target fraction, CD8+ T cell yield, on-target (NK cells) and off-target impurities (B cells, monocytes) of 4 independent selections. Boxes represent mean ± SD.

## Slide 5
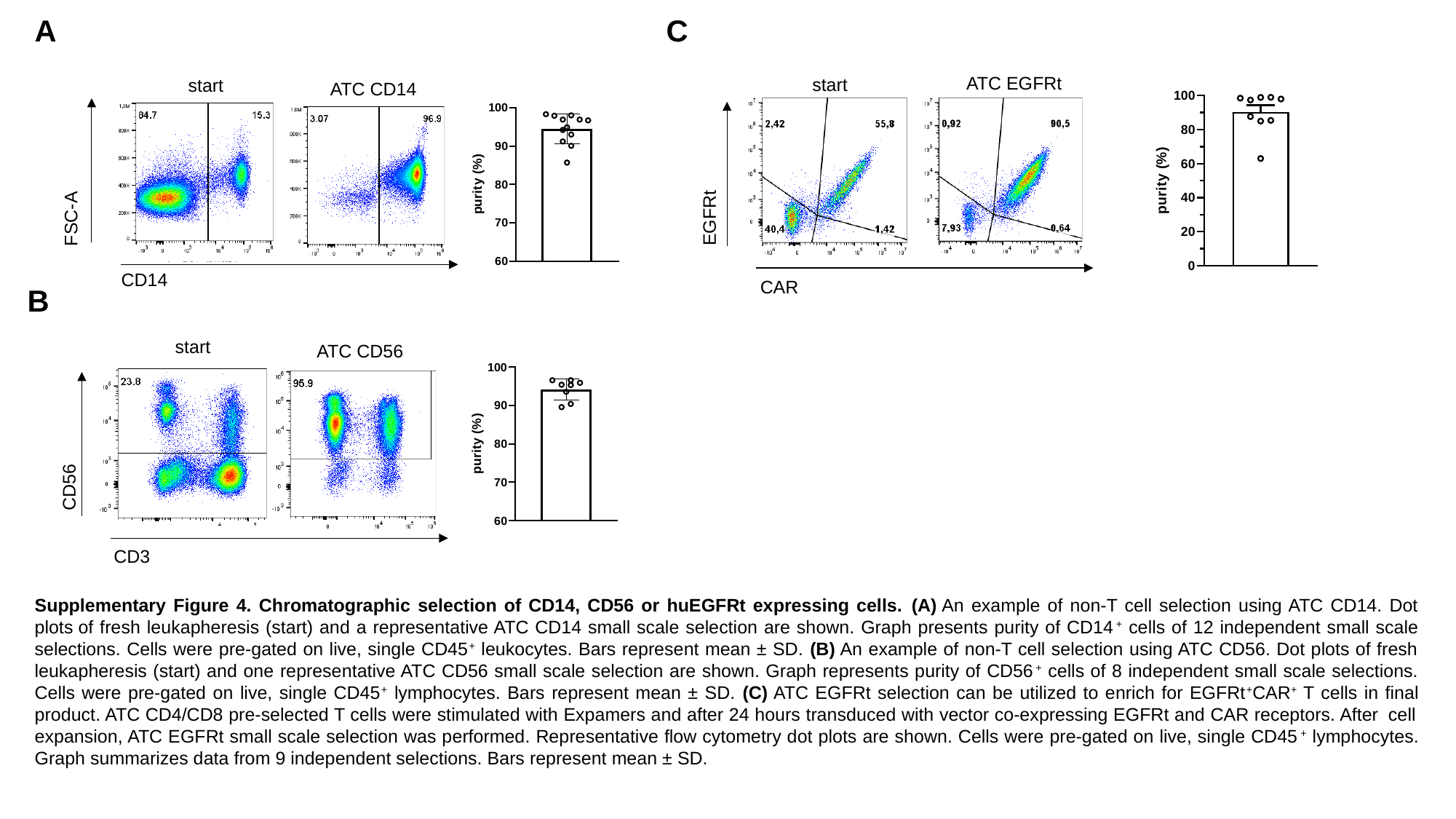

C
A
ATC EGFRt
start
start
ATC CD14
FSC-A
EGFRt
CD14
CAR
B
start
ATC CD56
CD56
CD3
Supplementary Figure 4. Chromatographic selection of CD14, CD56 or huEGFRt expressing cells. (A) An example of non-T cell selection using ATC CD14. Dot plots of fresh leukapheresis (start) and a representative ATC CD14 small scale selection are shown. Graph presents purity of CD14+ cells of 12 independent small scale selections. Cells were pre-gated on live, single CD45+ leukocytes. Bars represent mean ± SD. (B) An example of non-T cell selection using ATC CD56. Dot plots of fresh leukapheresis (start) and one representative ATC CD56 small scale selection are shown. Graph represents purity of CD56+ cells of 8 independent small scale selections. Cells were pre-gated on live, single CD45+ lymphocytes. Bars represent mean ± SD. (C) ATC EGFRt selection can be utilized to enrich for EGFRt+CAR+ T cells in final product. ATC CD4/CD8 pre-selected T cells were stimulated with Expamers and after 24 hours transduced with vector co-expressing EGFRt and CAR receptors. After  cell expansion, ATC EGFRt small scale selection was performed. Representative flow cytometry dot plots are shown. Cells were pre-gated on live, single CD45+ lymphocytes. Graph summarizes data from 9 independent selections. Bars represent mean ± SD.

## Slide 6
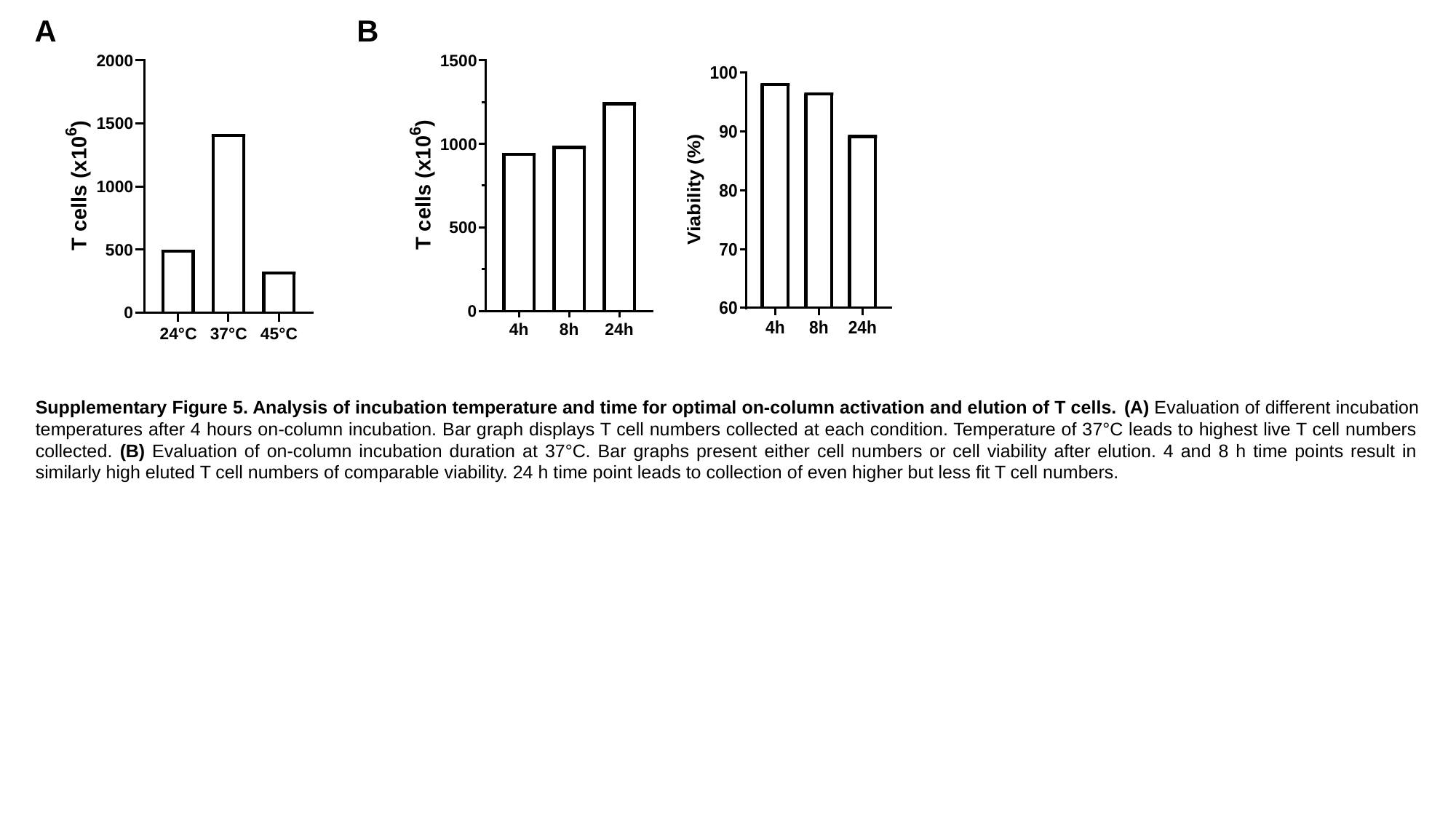

A
B
Supplementary Figure 5. Analysis of incubation temperature and time for optimal on-column activation and elution of T cells. (A) Evaluation of different incubation temperatures after 4 hours on-column incubation. Bar graph displays T cell numbers collected at each condition. Temperature of 37°C leads to highest live T cell numbers collected. (B) Evaluation of on-column incubation duration at 37°C. Bar graphs present either cell numbers or cell viability after elution. 4 and 8 h time points result in similarly high eluted T cell numbers of comparable viability. 24 h time point leads to collection of even higher but less fit T cell numbers.

## Slide 7
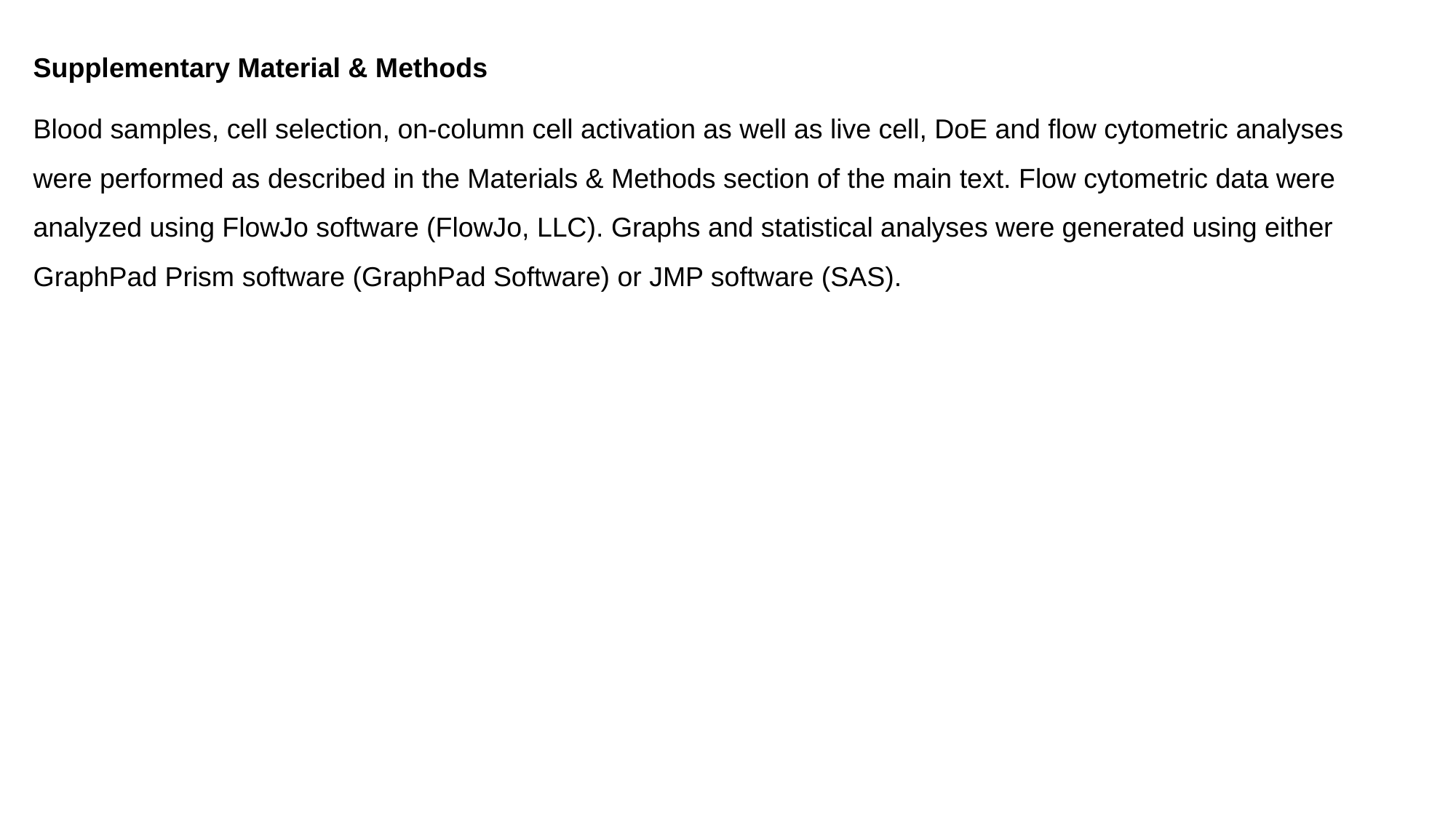

Supplementary Material & Methods
Blood samples, cell selection, on-column cell activation as well as live cell, DoE and flow cytometric analyses were performed as described in the Materials & Methods section of the main text. Flow cytometric data were analyzed using FlowJo software (FlowJo, LLC). Graphs and statistical analyses were generated using either GraphPad Prism software (GraphPad Software) or JMP software (SAS).
